# Cost effectiveness analysis of Year 2 of an elementary school-located influenza vaccination program–Results from a randomized controlled trial

**DOI:** 10.1186/s12913-015-1169-5

**Published:** 2015-11-16

**Authors:** Byung-Kwang Yoo, Sharon G. Humiston, Peter G. Szilagyi, Stanley J. Schaffer, Christine Long, Maureen Kolasa

**Affiliations:** 1 Department of Public Health Sciences, University of California Davis, School of Medicine, One Shields Ave. Medical Sciences 1C, Davis, CA 95616 USA; 2 Department of Pediatrics, Children’s Mercy Hospital, Kansas City, MO 64108 USA; 3 Department of Pediatrics, University of California Los Angeles (UCLA), 10833 Le Conte Avenue, Los Angeles, CA 90095 USA; 4 Department of Pediatrics, University of Rochester School of Medicine and Dentistry, 601 Elmwood Ave., Rochester, NY 14642 USA; 5 Center for Community Health of the University of Rochester, 46 Prince Street, Suite 1001, Rochester, NY 14607 USA; 6 Centers for Disease Control and Prevention, 1600 Clifton Rd., Atlanta, GA 30333 USA

**Keywords:** School-located vaccination program, Influenza vaccination, Community-based randomized controlled trial, Cost-effectiveness analysis, Incremental cost-effectiveness ratio

## Abstract

**Background:**

School-located vaccination against influenza (SLV-I) has the potential to improve current suboptimal influenza immunization coverage for U.S. school-aged children. However, little is known about SLV-I’s cost-effectiveness. The objective of this study is to establish the cost-effectiveness of SLV-I based on a two-year community-based randomized controlled trial (Year 1: 2009–2010 vaccination season, an unusual H1N1 pandemic influenza season, and Year 2: 2010–2011, a more typical influenza season).

**Methods:**

We performed a cost-effectiveness analysis on a two-year randomized controlled trial of a Western New York SLV-I program. SLV-I clinics were offered in 21 intervention elementary schools (Year 1 *n* = 9,027; Year 2 *n* = 9,145 children) with standard-of-care (no SLV-I) in control schools (Year 1 *n* = 4,534 (10 schools); Year 2 *n* = 4,796 children (11 schools)). We estimated the cost-per-vaccinated child, by dividing the incremental cost of the intervention by the incremental effectiveness (i.e., the number of additionally vaccinated students in intervention schools compared to control schools).

**Results:**

In Years 1 and 2, respectively, the effectiveness measure (proportion of children vaccinated) was 11.2 and 12.0 percentage points higher in intervention (40.7 % and 40.4 %) than control schools. In year 2, the cost-per-vaccinated child excluding vaccine purchase ($59.88 in 2010 US $) consisted of three component costs: (A) the school costs ($8.25); (B) the project coordination costs ($32.33); and (C) the vendor costs excluding vaccine purchase ($16.68), summed through Monte Carlo simulation. Compared to Year 1, the two component costs (A) and (C) decreased, while the component cost (B) increased in Year 2. The cost-per-vaccinated child, excluding vaccine purchase, was $59.73 (Year 1) and $59.88 (Year 2, statistically indistinguishable from Year 1), higher than the published cost of providing influenza vaccination in medical practices ($39.54). However, taking indirect costs (e.g., averted parental costs to visit medical practices) into account, vaccination was less costly in SLV-I ($23.96 in Year 1, $24.07 in Year 2) than in medical practices.

**Conclusions:**

Our two-year trial’s findings reinforced the evidence to support SLV-I as a potentially favorable system to increase childhood influenza vaccination rates in a cost-efficient way. Increased efficiencies in SLV-I are needed for a sustainable and scalable SLV-I program.

## Background

Seasonal influenza poses a substantial disease burden on children [1, 2]. In 2008, the United States Advisory Committee on Immunization Practices (ACIP), the American Academy of Pediatrics (AAP) and the American Academy of Family Physicians (AAFP) recommended universal annual seasonal influenza vaccination for all children aged 6 months to 18 years [3]. This recommendation was designed to protect children because influenza is a common cause of pediatric outpatient visits [4–6], hospitalizations [7–14] and deaths [15, 16]. Further, by reducing spread of disease, influenza vaccination for children can also reduce influenza-related morbidity and mortality among adults [17–19] as well as school absenteeism among children and teachers [20].

Despite these recommendations, vaccination coverage among school-aged children remains far below the most recent version of the Healthy People 2020 goal of 70 % coverage for all children [21, 22]. For the 2012–13 influenza season, only 58.6 % of 5–12 year olds and 42.5 % of 13–17 year olds received influenza vaccination [22]. Vaccinating all children within their primary care practices is a major challenge.

To attempt to increase vaccination coverage among school-aged children, some communities have implemented school-located vaccination against influenza (SLV-I) [23–25]. Cawley and associates conducted a systematic literature review and concluded that SLV-I is a promising option to achieve the expanded ACIP influenza vaccination recommendation [26]. They highlighted the need for well-controlled trials to establish the cost-effectiveness of specific influenza vaccination strategies. Some experts believe that “*the greatest need for future U.S*. [*SLV*-*I*] *program implementation is the development of a financially sustainable model that can be replicated annually on a national scale*” [23].

We conducted a community-based randomized controlled trial (RCT) of SLV-I among elementary school children in Monroe County, New York, during the fall seasons of 2009 and 2010 to examine the effectiveness (i.e., improvement in influenza vaccination rates), cost, and cost-effectiveness of SLV-I. The demonstration project’s design and effectiveness for Years 1 and 2 have been reported [27]. In addition, we have reported the project’s cost-effectiveness for Year 1 [28], which was the 2009 H1N1 pandemic year, and therefore unusual [27–29]. Analyzing the project’s Year 2 cost-effectiveness, a more typical influenza season than Year 1, could yield unique contributions to the literature. Specifically, the SLV-I program’s Year 2 might have been *more* efficient than Year 1 because of increased staff efficiency or program improvements (e.g., due to low utilization of a second clinic at each school in Year 1, only one clinic per school was offered in Year 2). Conversely, Year 2 might have been *less* efficient than Year 1 because of the parent demand in Year 1 being increased partly by the H1N1 influenza pandemic.

This paper reports on the Year 2 cost and cost-effectiveness of this SLV-I model, and compares these with corresponding values for Year 1. Our hypotheses were that the average cost-per-vaccinated child in SLV-I during Year 2 would be (1) lower than that observed in Year 1 and (2) comparable or lower than that observed in medical practices [30], when accounting for the costs to deliver vaccines and parents’ costs to visit medical practices for their child’s influenza vaccination. Our supplemental hypothesis was that in a Year 2 cost-effectiveness analysis, SLV-I could be cost-saving for society compared to “no vaccination.”

## Methods

The methods for data collection and analysis in Year 2 followed those in Year 1 [27, 28]. The study designs of Years 1 and 2 of this study were the same, with randomization of elementary schools into 21 intervention (SLV-I) schools (9027 students in Year 1; 9145 students in Year 2) and control schools (10 schools, 4534 students in Year 1; 11 schools, 4796 students in Year 2) (Table 1). Randomization assignments from Year 1 generally remained constant in Year 2 [27]. Based on the New York State Immunization Information System data, influenza vaccine coverage in 2008 (prior to Year 1) was similar for students in SLV-I and control schools (*P* > 0.2) [27]. The number of vaccination clinics at each school (two-per-school in Year 1; one-per-school in Year 2) differed by year. To make a fair comparison between the costs of the program in Years 1 and 2, we did not include vaccinations delivered at the second in-school clinic in Year 1. Our cost effectiveness analysis models derived all of the effectiveness parameters and most of the cost parameters from our community-based RCT (the Trial Registration Number for the RCT is ClinicalTrials.govNCT01224301). The Research Subjects Review Boards of the University of Rochester and the Monroe County Department of Public Health approved this study, including the informed consent procedure, in 2009.)

**Table 1:** Vaccination rates in school-located seasonal influenza vaccination (SLV-I) schools and control schools during Year 1 (2009–2010 season) and Year 2 (2010–2011 season)

	Year 1 (2009–2010)^a^					Year 2 (2010–2011)				
	SLV-I schools		Control schools		Difference between SLV-I and Control^b^	SLV-I schools		Control schools		Difference between SLV-I and Control^b^
	Students	% Total Students	Students	% Total Students		Students	% Total Students	Students	% Total Students	
TOTAL STUDENTS	9,027	100 %	4,534	100 %	0 %	9,145	100 %	4,796	100 %	0 %
Not vaccinated	5,356	59.3 %	3,195	70.5 %	−11.2 %	6,486	59.6 %	3,432	71.6 %	−12.0 %
Total vaccinated	3,671	40.7 %*	1,339	29.5 %*	11.2 %^d***^	3,698	40.4 %**	1,364	28.4 %**	12.0 %^d***^
Vaccinated elsewhere^c^	2,474	27.4 %	1,339	29.5 %	−2.1 %	2,659	29.1 %	1,364	28.4 %	0.6 %
Vaccinated at SLV-I	1,197	13.3 %^c^	n/a	n/a	13.3 %	1,039	11.4 %	n/a	n/a	11.4 %

n/a: not applicable

a: Only first clinics at each SLV-I school (excluding the vaccinated children at the second vaccination clinics (2.0 % among all students in Year 1))

b: The differences in the proportion between the column of “SLV-I schools (% Total Students)” and “Control schools (% Total Students)”

c: Identified as receiving at least the 1st dose outside the SLV-I schools in the New York State Immunization Information System (NYSIIS)

d: Primary effectiveness measure included in our cost-effectiveness analysis model

*: 40.7 % was significantly greater than 29.5 % (*p*-value < 0.0001) in Year 1

**: 40.4 % was significantly greater than 28.4 % (*p*-value < 0.0001) in Year 2

***: Primary effectiveness: 11.2 % in Year 1 was marginally smaller than 12.0 % in Year 2 (*p*-value = 0.092)

SLV-I clinics were held between 11/03/09 and 11/20/09 in Year 1 and between 11/2/2010 and 11/18/2010 in Year 2. A vaccination vendor administered either live attenuated (LAIV) or inactivated (TIV) seasonal influenza vaccines. Influenza vaccinations administered in the SLV-I clinics were recorded in the mass vaccinator’s database and analyzed in this study. Influenza vaccination rates outside the SLV-I clinics for both intervention and control schools were assessed by review of New York State Immunization Information System (NYIIS).

### Cost-effectiveness analysis (CEA)

We estimated the incremental cost-effectiveness ratio (ICER) by dividing the incremental cost (i.e., the difference in cost of vaccination in intervention schools minus the cost of vaccination in control schools (i.e., a reference group)) by the incremental effectiveness (i.e., the number of additionally vaccinated students in intervention schools compared to control schools). Both effectiveness and cost measures are defined below.

### Effectiveness measure

We defined effectiveness as the difference between the proportion of students who received ≥1 seasonal influenza vaccine anywhere (either at school or elsewhere) among students enrolled in intervention vs. control schools. This broad effectiveness measure was used because the intervention’s communication activities could have motivated parents to have their children vaccinated at either a medical practice or their schools.

### Cost measures

We calculated all costs in 2010 US $, adjusting with the consumer price index (CPI) when needed [31]. We estimated three “program costs” incurred by this study: (A) school costs, (B) project coordination costs, and (C) vendor costs, as well as two indirect costs averted by having the SLV-I program: (D) averted parents’ costs (i.e., costs to visit medical practices for a child’s influenza vaccination) and (E) costs averted by disease prevention (i.e., both reduced influenza-related medical costs among all household members and reduced loss of parental productivity related to caring for a sick child). “Net Cost” was defined as “Program costs” (A + B + C) less averted parent costs (D). “Societal Cost” was defined as “Program costs” (A + B + C) less costs averted by influenza prevention (E).

### Program cost components

Component A (school cost) included non-labor material cost (e.g., supplies and expenses associated with distributing information to parents) and labor costs. We calculated labor cost by multiplying the self-reported school staff hours by the national mean wage of a relevant job category as of May 2010 [32]. Component B (project coordination cost) included the cost incurred by coordinating activities, but excluded research and evaluation costs.

Component C (vendor costs) comprised the vendor’s labor (C_1_) and material costs, including broad items such as vaccine purchase, the refrigerator for vaccines and supplies. The vendor’s vaccine purchase costs were modeled in two ways. Our primary analysis, from the societal perspective, assigned a vaccine purchase cost (C_2_) of $12.06/dose for Year 2, which is the weighted average prices of federal Vaccine-For-Children (VFC) doses ($10.94 per dose; 52 % of all doses) and non-VFC doses ($13.26; 48 %) as of May 2010, listed on the CDC website [33]. These prices assumed that 80 % and 20 % of doses administered in this demonstration project were TIV and LAIV, respectively, in each year, as was observed in this trial’s Year 1. [28] Our supplemental analysis, assuming that VFC doses were free, was performed from the alternative perspective of school districts, health departments, and insurers (we included administration costs for private and VFC vaccine). In accordance with the Year 1 trail’s observation, the Year 2 analysis assumed that 52 % of students were VFC-eligible and, therefore, there was no charge to the vendor or families for their vaccine. Consequently, the cost item (C_3_) is the weighted average of VFC-dose ($0 per dose; 52 % of all doses) and non-VFC dose ($13.26; 48 %). However, it should be noted that these vaccine doses were not “free” from a societal perspective. Similarly, the vaccine prices for Year 1 were cited from the CDC website as of May 2009 [34].

### Components D and E

The Components D and E estimated the averted cost for a household with a child protected by influenza vaccine, compared to a household with an unvaccinated child. These cost estimates were assumed to be constant across two years when expressed in 2010 US $.

Averted parent costs (D) were derived previously [28] and included the cost of parents’ time [35–37] (two hours at the national median hourly wage of $21.25) and transportation ($6.42) [38]. Because some children would have had a medical visit to their primary care practice for reasons other than influenza vaccination during the influenza vaccination season, we took this into account. The sum of these averted costs ($21.25*2 h + $6.42 = $48.92) was discounted by 27.1 % (to $35.66) because the Medical Expenditure Panel Survey (MEPS) [39] showed that 27.1 % of children aged 7 to 12 years had ≥1 primary care visit between October and January (corresponding to typical influenza vaccination season). Also, these costs (D) do not include any medical expenditure, either paid by parents or incurred at medical practices.

The costs averted by disease prevention (component E) were derived from the literature. Based on findings from a large controlled clinical SLV-I trial, Schmier et al. estimated the averted medical expenditure as $121.46 and the averted productivity loss as $88.99 (both adjusted to 2010 dollars) per household, comparing the average costs per household between intervention and control schools during an influenza season [40]. The former averted medical expenditure represents the difference in direct influenza disease costs between intervention and control schools, including the costs for outpatient, emergency department, hospitalization, prescribed medication and over-the-counter medication [40]. The latter averted productivity loss accounted for the forgone income of a parent who was absent from work to take care of his/her influenza-infected child [40].

In the Schmier study [40], the average costs averted by disease prevention (component E) were $210.45 (=$121.46 + $88.99) per household comparing the intervention households (with a 47 % child vaccination rate) and the control households (with a 2 % child vaccination rate). On the other hand, because our study’s Component E represents the per-household averted cost comparing between households with a 100 % child vaccination rate and those with a 0 % child vaccination rate, the component E is expected to be at least $210.45. However, since the composition and household size were not assessed in our study unlike the Schmier study (i.e., 2.59 children and 2.0 adults per household), we made a conservative estimate for the component E as $210.45.

### Managing uncertainty

To address the uncertainty of the cost parameters in our model, we conducted a Monte Carlo simulation in summing the component costs, (A), (B) and (C_1_), to estimate subtotal costs and total costs. We defined a triangular distribution with modal, minimum and maximum values for each component cost. The modal value was assumed to be equal to the overall mean cost of all SLV-I schools. The minimum and the maximum values, for the components (A) and (C_1_), were determined among the eleven data points from the individual SLV-I schools, according to our Year 1 analysis, since school-related costs appeared to vary widely by school [28]. Regarding the component B (project coordination cost), we used 4 data points derived from 4 groups of schools based on the geographic area (urban/rural) and the intensity of an intervention outreach activity (high/low). The mean and the 95 % confidence interval (CI) of the simulation results with 10,000 iterations were reported, where 95 % CI was assumed to be equal to the range of the 2.5th and 97.5th quantiles of the iterations.

### Comparison with practice-located vaccination

We determined that SLV-I would be less costly than practice-located vaccination if the estimated cost-per-vaccinated child for SLV-I were below the median/mean ($22.17/$39.54 in 2010 US $) cost-per-vaccinated child for practice-located influenza vaccination as reported in our past study [28, 30]. For this comparison, we excluded the vaccine purchase cost from both SLV-I costs and medical practices’ costs [28, 30]. Finally, we subtracted the averted parent’s costs (Component D = $35.66) from the program costs of the SLV-I program.

## Results

### Effectiveness measures

The overall influenza vaccination rates (including vaccinations received in schools and at provider’s offices) in Years 1 and 2 were 40.7 % and 40.4 %, respectively, among children attending SLV-I schools compared to 29.5 % and 28.4 %, respectively, among those attending control schools (Table 1). Thus, the net effects of SLV-I was an 11.2 percentage point higher vaccination rates in Year 1 and a 12.0 percentage point higher vaccination rate in Year 2, suggesting a substantial impact of SLV-I in both the H1N1 Year 1 and the more typical seasonal influenza season in Year 2. These overall influenza vaccination rates of the SLV-I schools were slightly lower than among those aged 5–12 years enrolled in two nationally representative studies (i.e., National Health Interview Survey (NHIS) and National Immunization Survey (NIS)), e.g., 41.3 %-42.2 % during Year 1 and 45.9 %-54.7 % during Year 2 [41].

### Cost measures

**Table 2:** Cost-effectiveness analysis of school-located seasonal influenza vaccination (SLV-I) during the 2009–2010 season (Year 1)^a^ and the 2010–2011 season (Year 2) (all 2010 US $)^b^; Unit is incremental cost-effectiveness ratio (ICER)^c^ [$-per-incremental-student-vaccinated in the school-located seasonal influenza vaccination (SLV-I), compared to control schools]

	Year 1^a^	Year 2
COMPONENT COSTS^d^		
(A) School cost^e^	$9.92	$8.25
	($6.41, $14.75)^k^	($5.33, $12.26)^k^
(B) Project coordination cost^f^	$25.35	$32.33
	($16.37, $37.65)^k^	($20.87, $48.02)^k^
(C) Vendor cost from the societal perspective (=C_1_+ C_2_)	$33.99	$28.74
	($26.23, $44.76)^k^	($22.83, $36.84)^k^
(C_1_) Vaccine administration^g^	$21.91	$16.68
	($14.15, $32.68)^k^	($10.77, $24.78)^k^
(C_2_) vaccine purchase from the societal perspective (VFC dose = $10.76 (Year 1) $10.94 (Year 2)^h^	$12.08	$12.06
(C_3_) Vaccine purchase from the alternative perspective of school districts, health departments, and insurers (VFC dose = $0)^h^	$6.48	$6.37
(D) Averted parents’ costs (i.e., to visit medical practices for a child’s influenza vaccination)^i^	$35.66	$35.66
SUBTOTAL COSTS^j^		
Subtotal Net Cost 1: (A + B)-(D)	$1.15^m^	$6.64^m^
	(−$7.46, $10.31)^l^	(−$3.93, $17.93)
Subtotal Program Cost 2: (A + B + C_1_)	$59.73	$59.88
	($48.26, $71.73)^l^	($47.69, $72.74)^l^
Subtotal Program Cost 3: (A + B + C_1_ + C_3_)	$66.19	$66.06
	($54.94, $78.13)^l^	($53.94, $78.69)^l^
TOTAL COSTS^h^		
Total Program Cost 1 from the societal perspective: (A + B + C)	$71.78	$71.74
	($60.32, $83.77)^l^	($59.75, $84.47)^l^
Total Net Cost 2 to compare with influenza vaccination in private pediatric practices: (A + B + C_1_)-(D)	$23.96^n^	$24.07^n^
	($12.79, $35.99)^l^	($12.08, $37.00)^l^
Total Net Cost 3 from the societal perspective accounting for averted parents’ costs: (A + B + C)-(D)	$36.16	$36.01
	($24.82, $48.13)^l^	($23.93, $48.76)^l^

a: Only first clinics at each SLV-I school (not including the vaccinated children at the second vaccination clinics (2.0 % among all students) in Year 1)

b: All cost estimates were adjusted to 2010 U.S. dollar values with the consumer price index when needed [31]

c: Incremental cost-effectiveness ratio (ICER) was estimated by dividing the incremental cost (i.e., the difference in cost of vaccination in intervention schools minus the cost of vaccination in control schools (i.e., a reference group)) by the incremental effectiveness (i.e., the number of additionally vaccinated students in intervention schools compared to control schools). In control schools, some students were vaccinated outside schools (e.g., medical practices), as summarized in Table 1

d: The values within the parentheses in the rows for COMPONENT COSTS indicate the triangular distributions defined by the modal value, (minimum value and maximum value). The modal value was assumed to be equal to the overall mean of the SLV-I schools. The minimum and the maximum values were determined among the eleven mean cost estimates from eleven SLV-I schools, following our Year 1 analysis [28]

e: Composed of material cost and labor cost. Material cost includes information distribution (to parents) costs such as paper, mailing, and phone. Labor cost was calculated through “the time spent for the project by school staffs” multiplied by “category-specific hourly wage (national average)” as of May 2009 [58] and May 2010 [32] for Year 1 and Year 2, respectively

f: Time cost for collection of consent forms, and meeting with school staffs, and vendors. Evaluation research cost was excluded

g: Composed of the vendor’s labor and material costs, including broad items such as the refrigerator for vaccines and supplies

h: 52 % of the administered doses that were provided by Vaccine-for-children (VFC) for free. From a societal perspective, we assigned $10.76 (Year 1) and $10.94 (Year 2) per dose as the vaccine purchase cost, which is the weighted average prices of TIV (80 % of doses administered in this demonstration) and LAIV (20 %) listed in the CDC website as of May 2009 [34] and May 2010 [33] for Year 1 and Year 2, respectively

i: This (D) averted parents’ costs indicate the costs to visit medical practices for a child’s influenza vaccination, consisting of parents’ time cost [35–37] and transportation cost [38]. These costs do not include any medical expenditure, either paid by parent or incurred at medical practices

j: The values within the parentheses in the rows for subtotal costs and total costs indicate the mean and 95 % confidence interval of Monte Carlo Simulation results (10,000 iterations) using the distributions defined in the rows for component costs. The 95 % confidence interval was assumed to be equal to the range of the 2.5th and 97.5th quantiles of the iterations

k: The minimum and the maximum values, for the components (A) and (C_1_), were determined among the eleven data points from the individual SLV-I schools. Regarding the component (B), we used 4 data points derived from 4 groups of schools based on the geographic area (urban/rural) and the intensity of an intervention outreach activity (high/low)

l: 95 % confidence interval values (under a Monte Carlo Simulation) are in parentheses

m: Below the lower limit of the cost range ($11.59, $17.38) [per child vaccinated] in the reminder program (using letters and/or automated telephone message) estimated by Lieu et al. [59]

n: Falls between the 25th percentile ($13.88) and the median/mean ($22.17/$39.54) cost [per dose] for providing influenza vaccination in private pediatric practices estimated by Yoo et al. [28, 30]

The number of students vaccinated in SLV-I schools (Table 1) was used as the denominator for the cost measure estimation and cost effectiveness analysis. The project coordination cost (B) per vaccinated-child was higher in Year 2 than Year 1, ($32.33 vs. $25.35), more than offsetting increased efficiencies in other costs (Table 2). In contrast, school costs (A) per vaccinated-child were lower in Year 2 than Year 1, ($8.25 vs. $9.92) as were the vendor costs (C) per vaccinated-child ($28.74 vs. $33.99). Since the vaccine purchase costs (C_2_) could vary substantially depending on the proportion of vaccine-for-children (VFC) eligible children, we made separate costs estimates: (i) including the vaccine administration cost only (C_1_) and (ii) additionally including the vaccine purchase cost (C_2_).

### Cost effectiveness analysis (CEA)

#### Comparison between years 1 and 2

The Subtotal and Total costs in Table 2 present the CEA results, i.e., ICER estimates based on the Monte Carlo simulation, that enable us to compare the overall SLV-I program performance between Years 1 and 2. Our results showed that the ICER estimates for all types of Subtotal and Total costs were not significantly different between Years 1 and 2. Thus, our first hypothesis -- that the average cost per vaccinated child in SLV-I schools would be lower during Year 2 than Year 1–was not supported. Note that despite differences in point (mean) estimates, there was marked overlap in the 95 % CIs between Years 1 and 2.^1^

#### Comparison with practice-located vaccination

When we consider only program costs, we find that SLV-I costs were higher than costs observed in medical practices. Specifically, based on the Subtotal Program Cost 2 (based only on Components A + B + C_1_), the mean ICER estimates were $59.73 in Year 1 and $59.88 in Year 2. These mean estimates and the lower bounds of 95 % CI $48.26 in Year 1 and $47.69 in Year 2) were all higher than previously reported corresponding costs in medical practices (median/mean = $22.17/$39.54) [28, 30].

When also considering indirect averted parent costs (D) (Total Net Cost 2: Components A + B + C_1_–D), the mean ICER estimates would decline to $23.96 in Year 1 and $24.07 in Year 2. These mean ICER estimates in both years fall between the 25th percentile ($13.88) and the mean ($39.54) cost for providing influenza vaccination in medical practices [28, 30], supporting our second hypothesis that SLV-I costs would be comparable or lower than practice-based influenza vaccination costs when including indirect averted parent costs (Component D).

When including indirect costs averted by disease prevention (E), the mean ICER estimate for Year 2 became negative ((A + B + C)–E = $71.74 - $210.45 = −$-138.71) (results not shown in Table 2). Negative ICER values indicate that SLV-I is cost saving compared to “no vaccination” from a broader societal perspective.

Finally, we performed two sets of break-even analyses for CEA in Year 2 only: (1) Based on the Total Net Cost 3 [(program costs − parent costs) = (A + B + C − D)], we estimated the threshold level of in-school vaccination necessary for SLV-I to be cost-saving to society, even if averted costs due to medical expenditures and lost productivity were not included. This would be achieved if the in-school net vaccination rate were to increase from the actual level (11.4 % in Year 2) to at least 24.0 %, assuming no change in vaccination rates ‘elsewhere.’ (2) Based on the Subtotal Program Cost 2 (A + B + C_1_), we estimated the level of in-school vaccination necessary for mean cost in SLV programs and medical practices to be equivalent, even if averted parents’ costs to visit a medical practice (D) were not included. This would be achieved if the in-school vaccination rate were at least 17.6 %, assuming no change in vaccination rates ‘elsewhere.’

## Discussion

We had hypothesized that the SLV-I program would become more efficient in Year 2 of implementation, but found that the costs and cost-effectiveness of SLV-I were comparable in Year 2, a routine seasonal influenza vaccination year, compared to Year 1, the year of pandemic H1N1. Project coordination costs (Component B) were higher in Year 2 than 1, more than offsetting improved efficiencies in other costs. A detailed comparison between the two years offers useful policy implications for SLV-I and can help set future goals to improve the overall cost-effectiveness of SLV-I during a more typical season. Additionally, analyses of both Years 1 and 2 showed that SLV-I could be cost-saving to society, compared to “no vaccination,” if savings from the increase in disease prevention under the SLV program (Component E) were included.

### Comparison with vaccination in medical practices

When considering program costs alone, the cost to vaccinate a child in SLV-I was higher than the previously calculated cost to vaccinate a child in primary care practices in the same community. When considering program plus averted parent costs for transportation and time lost from work (i.e., cost component (D)), the cost to vaccinate a child in SLV-I was comparable or lower than that in primary care practices. Communities interested in implementing SLV-I will naturally focus on the difference between program costs and revenues received from SLV-I in determining feasibility and sustainability of SLV-I.

### Comparison between year 1 and year 2

Project coordination costs (Component B): The major reason why the SLV-I project was not more cost-effective in Year 2 than Year 1 was that the project coordination costs (Component B) increased during Year 2, more than offsetting decreases in school and vendor costs (Components A and C). In Year 2 pre-season, an additional four weeks was available for planning leading to more time being devoted to the process. Variation in responsibility for some tasks in Years 1 and 2 among school staff and project administrative staff may have led to changes in Components A and B across years. Because of this, we also compared the sum of Components of A and B -- $35.27 (Year 1) was still lower than $40.58 (Year 2). Given the realities of busy school personnel and the reduction in school nurses nationwide, we believe that for SLV-I to be sustainable, work by school staff would need to be minimized, with the possible exception of a special pandemic season. Thus, it is possible that the costs noted in Year 2 may represent a more generalizable estimate. Regardless of the reasons for the high costs, it is clear that for SLIV to be sustainable and scalable, fieldwork costs outside of the school-based costs would need to be lower than we experienced. Further studies are needed to assess the degree to which fieldwork costs can be reduced, e.g., costs to coordinate five school-districts in our trial which imposed much higher burdens compared a single school-district trial [42].

### Comparison of year 2 trial with other studies

Our Year 2 intervention had a moderate impact on influenza vaccination uptake with an improvement of 12 percentage points (pp). This impact was higher than that found in other trials, such as those using text message reminders (3.7 pp) [43], mail reminders (6.5 pp) [44], and provider prompts (4.0 pp but statistically insignificant) [45].

The overall vaccination coverage rates found in our trial was in the range of rates noted in other studies. For example, SLV-I trials at the state level in Hawaii and the three-county level in Minnesota resulted in very high vaccination rates of 46 % [25] and 41 % [46], respectively; however these were not clinical trials, no control schools existed, and the papers did not report what percent of vaccinations were delivered in school versus in physician offices. In an SLV-I trial at nineteen elementary schools in California, coverage varied by school, with 26.9 %-46.6 % of children in each school receiving at least one dose of influenza vaccine with a large impact due to SLV-I; however in this setting control schools had virtually zero vaccination rates [47].

Unlike other SLV-I trials, our SLV-I trial billed insurers (or parents if insurance coverage was unknown) for vaccines and vaccine administration. We contracted with a for-profit vendor that delivered the in-school vaccinations, which were purchased through routine channels or obtained through VFC program. Consequently, the cost estimates from our study are greater than those in other SLV-I trials that did not include vaccine cost and\or the cost to bill insurance or Medicaid for vaccines or vaccine administration. The cost estimates in other studies in this section were all adjusted to 2010 US $ with medical care CPI [31]. For instance, our estimate of $59.88 per vaccinated-child was much lower than that by Kansagra et al. ($80.92) [48], but considerably higher than that by Schmier and colleagues ($6.98) [40], Hull and associates ($10.11) [46], Effler et al. ($15.66) [25], and Kemp and colleagues ($24.69) [49]. Among these five studies, only Kansagra et al. and Effler et al. reported the detailed cost items within the administration cost. Concerning the labor cost estimates, our estimate ($39.13 per vaccinated-child) was slightly larger than $33.17 estimated by Kansagra et al. [48], and much larger than $12.19 estimated by Effler et al. [25]. The latter lower estimate may have been partly due to economies of size of their large state-wide program, vaccinating 63,153 children after targeting all children aged 5–13 in Hawaii [25]. Additionally, the studies by Effler et al. [25] *or* Hull et al. [46] did not seek third party reimbursement, which was included as part of the vendor’s administrative cost (for the billing process) in our study. Parents were not billed for any fees in the study by Kemp et al. [49], although our cost estimates includes the vendor’s billing process costs for parents.

Extensive project staff time was needed to manage parents’ consent forms–all done on paper -- which included details of about patient insurance. More efficient consent systems could reduce future SLV-I program costs [50, 51].

Material costs incurred by schools and the project coordinators were $4.69 per vaccinated-child, which was similar to $5.72 (adjusted to 2010 US $ with medical care CPI) reported by Effler et al. [25]. However, our estimate of the vendor’s material cost, $13.43 per vaccinated-child, was much higher than that of $1.64 by Effler et al. [25]. This difference can be partly explained by our study’s broader cost definition including items such as the refrigerator for vaccines and non-medical supplies.

Other studies also have concluded that SLV-I may be cost saving to society, when considering broader indirect costs [40, 52]. Using secondary datasets only, White and associates estimated that group-based influenza vaccination was cost-saving, i.e., saving $6.40 and $55.82 per vaccination, as compared to individual vaccination at a medical practice and no-vaccination, respectively [52]. Schmier et al. analyzed their primary data to conclude that SLV-I is cost-saving to society, saving $170.31 on average among all households in intervention schools [40].

In summary, our study, based upon a real-world demonstration and including billing of third party payers, had higher program costs than most prior SLV-I studies, resulting in lower cost-effectiveness. Our findings regarding indirect parent or societal costs were in line with those of other studies.

### Potential limitations

There was uncertainty in cost estimates in Year 1 that may affect the comparison with Year 2. As discussed in the paper describing Year 1 [28], it is difficult to accurately allocate the fixed costs between first clinics and second clinics during Year 1 due to the limited available data.

Another limitation is the potentially limited generalizability of our estimates, which may have been affected by multiple factors. The effectiveness of SLV-I is sensitive to the proportion of local children vaccinated by medical practices prior to the school vaccine clinics. For instance, in an area where medical practices vaccinate a high proportion of children, a SLV-I program may have a smaller impact on vaccination coverage. Hence, our SLV-I effectiveness estimates are likely to be most applicable to other areas where the vaccination rates achieved by medical practices are similar to those in our study site, i.e., less than one-third of children were vaccinated pre-intervention. Second, since influenza vaccination rates may be influenced by a host of seasonal influenza factors (e.g., disease severity [53], vaccine availability [54], media coverage [55]) SLV-I vaccination coverage in school and, consequently, effectiveness estimates could differ from year to year.

Different methods were used to ascertain the vaccinated status between intervention schools (based on the vendor’s records and New York State Immunization Information System (NYSIIS)) and control schools (NYSIIS records only). This difference could affect the effectiveness measure within a year, but would not affect the comparison between Years 1 and 2.

Finally, we derived indirect costs from the literature, not from our trial. Since we utilized a national-level median hourly wage among working adults for estimating the indirect cost component D, averted parents’ costs, these estimates are expected to be reasonably generalizable. Since the magnitude of the other indirect component (E, costs saving from disease prevention) might be sensitive to the estimation methods and the seasons analyzed, component E-related results were not presented in Tables. Our analysis excluded some relevant, but unmeasured, indirect costs such as costs due to disruptions of the school day by SLV-I, and cost savings from decreased absenteeism.

### Policy implications

Our findings from a real-world demonstration project indicated that while SLV-I is effective in improving influenza vaccination rates in school-aged children, project coordination costs (Component B) remained high during a second project year. High project coordination costs (Component B), driven by a substantial amount of effort needed to obtain informed consent and to manage implementation of the project, more than offset lower school and vendor costs (Components A and C). Project coordination costs (Component B) would need to be reduced through strategies such as an efficient parent consent and communication system. Overall, the per-vaccinated child cost estimates of our SLV-I were higher than those in medical practices and also higher than typical reimbursement rates. While our current cost estimates favor SLV-I over medical practices when we account for averted parental costs to visit medical practices (Component D), such cost-savings to parents may not be considered by health systems responsible for SLV-I. Thus, while costs are not the only consideration in setting up and sustaining SLV-I [56, 57], the program costs for SLV-I should be lower than practice-located costs, or at least lower than or equal to reimbursement rates for SLV-I to be sustained.

Finally, achieving higher in-school vaccination coverage would improve cost-effectiveness. For example, in this study a net vaccination rate of 17.6 % (rather than 11.4 % found in Year 2) would lead to SLV-I cost estimates being lower than those in medical practices.

## Conclusions

Our two-year trial’s findings reinforced the evidence to support SLV-I as a potentially favorable system to increase childhood influenza vaccination rates in a cost-efficient way, but increased efficiencies in SLV-I are needed for a sustainable and scalable SLV-I program.

## Abbreviations

AAFP: American academy of family physicians
AAP: American academy of pediatrics
ACIP: advisory committee on immunization practices
CEA: cost-effectiveness analysis
CI: confidence interval
ICER: incremental cost-effectiveness ratio
LAIV: live attenuated influenza vaccine
NYIIS: New York State immunization information system
RCT: randomized controlled trial
SLV-I: school-located vaccination against influenza
TIV: trivalent influenza vaccine
VFC: vaccine-for-children

## Footnotes

1 For instance, using our primary effectiveness measure (i.e., columns labeled “vaccinated anywhere” in Table 2), the mean ICER estimate based on Total Program Cost 1 was $71.74 per incremental-student-vaccinated in Year 2, which was lower than $71.78 in Year 1. However, because the estimated 95 % CI in Year 2 ($59.75, $84.47) overlaps with that in Year 1 ($60.32, $83.77), there is no significant difference in Total Program Cost 1 between Years 1 and 2.
